# Inhibitory control in neuronal networks relies on the extracellular matrix integrity

**DOI:** 10.1007/s00018-021-03861-3

**Published:** 2021-06-15

**Authors:** Egor Dzyubenko, Michael Fleischer, Daniel Manrique-Castano, Mina Borbor, Christoph Kleinschnitz, Andreas Faissner, Dirk M. Hermann

**Affiliations:** 1 Department of Neurology and Center for Translational Neuro- and Behavioral Sciences (C-TNBS), University Hospital Essen, Hufelandstraße 55, 45122 Essen, Germany; 2 Department of Cell Morphology and Molecular Neurobiology, Faculty of Biology and Biotechnology, Ruhr University Bochum, Universitätsstraße 150, 44801 Bochum, Germany

**Keywords:** Neuronal network activity, ECM, Inhibitory synapse, E-I balance, Electrophysiology

## Abstract

Inhibitory control is essential for the regulation of neuronal network activity, where excitatory and inhibitory synapses can act synergistically, reciprocally, and antagonistically. Sustained excitation-inhibition (E-I) balance, therefore, relies on the orchestrated adjustment of excitatory and inhibitory synaptic strength. While growing evidence indicates that the brain’s extracellular matrix (ECM) is a crucial regulator of excitatory synapse plasticity, it remains unclear whether and how the ECM contributes to inhibitory control in neuronal networks. Here we studied the simultaneous changes in excitatory and inhibitory connectivity after ECM depletion. We demonstrate that the ECM supports the maintenance of E-I balance by retaining inhibitory connectivity. Quantification of synapses and super-resolution microscopy showed that depletion of the ECM in mature neuronal networks preferentially decreases the density of inhibitory synapses and the size of individual inhibitory postsynaptic scaffolds. The reduction of inhibitory synapse density is partially compensated by the homeostatically increasing synaptic strength via the reduction of presynaptic GABA_B_ receptors, as indicated by patch-clamp measurements and GABA_B_ receptor expression quantifications. However, both spiking and bursting activity in neuronal networks is increased after ECM depletion, as indicated by multi-electrode recordings. With computational modelling, we determined that ECM depletion reduces the inhibitory connectivity to an extent that the inhibitory synapse scaling does not fully compensate for the reduced inhibitory synapse density. Our results indicate that the brain’s ECM preserves the balanced state of neuronal networks by supporting inhibitory control via inhibitory synapse stabilization, which expands the current understanding of brain activity regulation.

**Supplementary Information:**

The online version contains supplementary material available at 10.1007/s00018-021-03861-3.

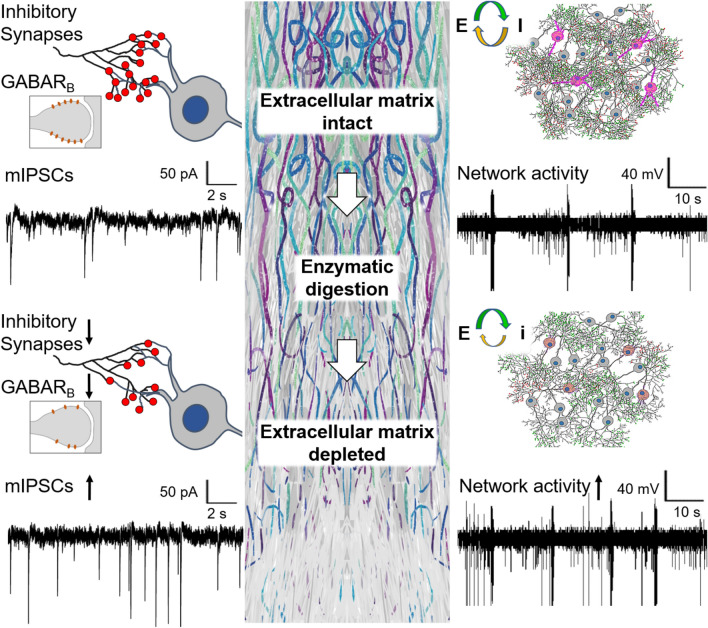

## Introduction

Neuronal activity is regulated through a dynamic balance of excitation and inhibition (E-I balance) [35] that requires coordinated plasticity of excitatory and inhibitory synapses [6, 69]. In the brain, excitatory and inhibitory synapses are regulated cooperatively [25, 60], and their modulation can be synergistic, reciprocal, and antagonistic. Over several years, experimental studies have gathered solid evidence on the plasticity of individual excitatory synapses, establishing the modulation of presynaptic neurotransmitter release and postsynaptic responsiveness to glutamate as key neural correlates for memory and learning [39]. The co-regulation of excitatory and inhibitory synapses is less studied. On the network level, scaling of inhibitory synapses is essential for homeostatic mechanisms maximizing information processing capacity in neuronal networks [45]. Computational modelling suggests that patterns of neuronal network activity are primarily determined by inhibitory connectivity [47]. Yet, while the understanding of inhibitory synapse regulation continuously evolves [13, 29], our knowledge about how inhibitory plasticity modulates neuronal network activity at the integrative level, remains limited.

The brain extracellular matrix (ECM) is a multicomponent macromolecular meshwork containing chondroitin sulfate carrying proteoglycans (CSPGs), which anchors to the neuronal surface via hyaluronic acid synthesizing enzymes [43, 59]. Neuronal activity induces the consolidation of ECM molecules [19] forming densely packed lattice-shaped layers around a subpopulation of neurons. These coatings, termed perineuronal nets (PNNs), support the fast-spiking properties of interneurons [16], regulate learning, and help to retain acquired memory [10]. In adulthood, CSPGs of PNNs and interstitial ECM restrict neuronal plasticity [11, 12, 20, 54]. A previous study [32] demonstrated that the lack of four major ECM proteoglycans (tenascin-C, tenascin-R, brevican, and neurocan) compromises PNN integrity and alters neuronal network development. In the present work, we explored how ECM depletion affects mature neuronal networks, in which earlier developmental stages were not augmented via genetic manipulation.

Enzymatic digestion of the brain ECM increases lateral mobility of excitatory glutamate receptors, alters short-term plasticity of excitatory synapses [28] and enhances neuronal network activity [7]. Thus, ECM integrity is essential for sustained synaptic signaling and neuronal circuit stability. Upon brain injury induced by ischemic stroke, the ECM is rapidly degraded within a few hours [36], and it stays partly decomposed after one week in lesion-surrounding brain areas [22], in which neuronal network activity is compromised [17, 44]. To understand how ECM alterations affect neuronal activity after brain injury, it is necessary to investigate the adjustment of neuronal networks at the integrative level after ECM depletion by bridging synaptic, neuronal, and network activity changes. Despite the link between ECM integrity and inhibitory interneuron function was previously demonstrated [4, 5, 67], the role of inhibitory synapse plasticity in controlling network activity after ECM depletion remained unknown.

Here we investigated the role of the brain ECM for stabilizing excitatory and inhibitory synapses and balancing neuronal network activity. We enzymatically degraded hyaluronic acid and CSPGs in the extracellular space and combined structural and functional readouts for studying neuronal connectivity and activity at the synapse and network level. By using data-driven computer simulations, we explored the influence of synaptic strength and connectivity changes on the activity of neuronal networks.

## Materials and methods

### Legal issues and animal housing

Experimental procedures were approved by the local government (Bezirksregierung Düsseldorf) and conducted in accordance with European Union (Directive 2010/63/EU) guidelines for the care and use of laboratory animals. C57BL/6j mice (Envigo, Indianapolis, IN, U.S.A.) were kept in groups of 5 animals/cage in a regular inverse 12 h light–dark cycle and access to food and water ad libitum. All efforts were made to reduce the number of animals in the experiments.

### Cell cultures

Primary cultures of neurons and astrocytes were prepared as described previously [31, 33]. Hippocampal neurons were obtained at embryonic day 15 (E15) and cortical astrocytes were obtained at postnatal day 1 (P1) from male and female mice. Neurons were supported by astrocyte monolayers cultivated on cell culture inserts with a permeable membrane (Fig. S1A), allowing for the long-term maturation of neuronal networks. We plated 50,000 neurons in a 50 µl droplet onto pre-treated glass coverslips or MEA chips. Inserts containing 50,000 astrocytes were combined with neuronal cultures on the same day. The cultures were maintained in Neurobasal medium (21103049, ThermoFisher, Waltham, MA, U.S.A.) supplemented with 2 mM L-glutamine (25030081, ThermoFisher), 1% v/v B27 (A3582801, ThermoFisher) and 1% v/v SM1 (05711, STEMCELL Technologies, Vancouver, Canada). We changed half of the medium weekly to keep the pH around 6.5. All in vitro experiments were performed on fully mature neurons after 21–28 days of cultivation (Figs. S1 and S2). The mature networks consisted of ~ 67% principal excitatory neurons and ~ 33% inhibitory interneurons, which is slightly higher than 20–25% in vivo [38, 66]. Virtually all inhibitory cells were parvalbumin-containing, and the percentage of other interneuron types could be therefore neglected [21]. The evolution of neuronal networks in vitro was characterized by coherent maturation of synaptic connectivity and ECM expression, partially resembling neuronal circuit development in vivo [14]. Synaptic connectivity of neuronal networks in vitro established randomly, mimicking the organization of cortical networks [3, 61]. During cultivation, synapse density increased until maturity was reached after 21 days in vitro (DIV) (Fig. S1B, C). The establishment of network connectivity correlated with the expression of PNNs, the condensed ECM layers characteristic for the mature neuronal cultures (Fig. S1B, D). Network activity in neuronal cultures evolved accordingly, changing from the high-frequency random spiking at 7 DIV followed by the low activity period at 14 DIV and the regular bursting pattern at 21 DIV (Fig. S1E). At later time points (i.e., 35 DIV) synapse density, PNN number, and network activity were stabilized and did not change. The mature neuronal cultures contained 34 ± 6.3% (mean ± s.e.m.) inhibitory interneurons (Fig. S2).

### ECM depletion

We followed an established approach for enzymatic ECM digestion [7, 54, 56]. The treatment solutions were labelled in a non-descriptive manner (i.e. A, B, C), making the experimenter blinded during the treatment, data acquisition, and data analysis. Cell cultures were incubated with 500 mU/ml ChABC (C3667, Sigma-Aldrich, Taufkirchen, Germany) or 500 U/ml hyaluronidase (HYase, H4272, Sigma-Aldrich) for 16 h. For ECM depletion in vivo, 500 mU ChABC or 500 U HYase were dissolved in 2 µl of 0.1 M PBS and delivered via a single stereotactic (bregma 0, left 3 mm, deep 1 mm) intracortical injection. At 16 h post-injection, animals were sacrificed and brains were processed for further analysis. Control animals were treated with a vehicle (0.1 M PBS). In our hands, the two enzymes were equally efficient for ECM digestion and specifically targeted the neuronal cell culture compartment, while no alterations in glial cells were detected (Fig. S3). Moreover, ChABC and HYase induced identical changes in synapse density and network activity (Fig. S4).

### Immunolabelling procedures

For immunohistochemistry, brains were perfused with 4% w/v paraformaldehyde (PFA) and post-fixed for 12 h in 4% w/v PFA. 40 µm coronal free-floating sections were obtained from the bregma level. For immunocytochemistry, cell cultures were fixed with 4% w/v PFA for 10 min at room temperature. Synaptic proteins were detected mouse anti-PSD95 (1:500, MAB1598, Millipore, Burlington, MA, U.S.A.), guinea pig anti-VGLUT1 (1:500, 135304, Synaptic Systems, Goettingen, Germany), mouse anti-gephyrin (1:500, 147011, Synaptic Systems) and guinea pig anti-VGAT (1:500, 131103, Synaptic Systems) antibodies. For GABA receptor quantification, GABA_A_ and GABA_B_ receptors were labelled with chicken anti-GABA_A_ γ2 (1:500, 224006, Synaptic Systems) and rabbit anti-GABA_B_ (1:500, 322102, Synaptic Systems) antibodies. To characterize the ECM expression, we applied biotinylated WFA (1:100, B-1355, Vector Laboratories, Burlingame, USA), biotinylated hyaluronan binding protein (1:100, 400763, AMS Biotechnology, Frankfurt, Germany), rabbit anti-aggrecan antibody (1:500, AB1031, Millipore) and rat anti-473HD antibody (1:100, produced by the group of Prof. Andreas Faissner, Bochum, Germany; [72]). Neuronal types were identified using rabbit anti-GABA (1:2000, A2052, Sigma-Aldrich, chicken anti-NeuN (1:300, ABN91, Millipore, mouse anti-neurofilament M (1:500, 171231, Synaptic Systems and rabbit anti-Kv3.1b (1:1000, APC-014, Alomone Labs, Jerusalem, Israel antibodies. Rat anti-glial acidic fibrillary protein (GFAP; 1:1000, 13–0300, ThermoFisher, mouse anti-β-catenin (1:500, ab19381, Abcam, Cambridge, UK and rabbit anti-connexin 43 (1:500, 3512, Cell Signaling Technologies, Frankfurt, Germany antibodies were used as astroglial markers. For fluorescence detection, secondary antibodies conjugated to Alexa or Atto dyes were used. Nuclei were counterlabeled with DAPI (1:1000, D1306, ThermoFisher).

### Low-resolution microscopy for basic quantifications

For basic quantifications of cell density and marker proteins expression following immunohistochemistry/ immunocytochemistry, four 425.1 × 425.1 μm regions of interest (ROIs) per condition per experiment were selected at random positions for cell culture specimens. In brain sections, the ROIs were positioned in the left and right cerebral somatosensory cortex layers 3–5. Single plane micrographs were obtained using the Carl Zeiss LSM710 confocal microscope (20 × Plan Apochromat objective, NA 0.8).

### Total expression of GABA receptors

The total expression of GABA receptors on the neuronal surface was investigated in vitro by Western blot. Membrane proteins were extracted using the Mem-PER Plus Membrane Protein Extraction Kit (89842, ThermoFisher) and separated by sodium dodecyl sulfate polyacrylamide gel electrophoresis (SDS-PAGE) on 1 mm 8% polyacrylamide gels. To avoid protein aggregation the samples were not heated. The proteins were transferred onto nitrocellulose membranes using the Trans-Blot Turbo Transfer System (Biorad, Hercules, CA, U.S.A.) mixed molecular weight program, followed by pre-blocking in 3% bovine serum albumin (BSA) for 1 h. The membranes were incubated with primary chicken anti-GABA_A_ γ2 (1:1000, 224006, Synaptic Systems), rabbit anti-GABA_B_ (1:1000, 322102, Synaptic Systems) and mouse anti-synaptotagmin-1 (1:1000, 105011, Synaptic Systems) antibodies for 72 h at 4 °C. Secondary antibodies were applied stepwise, and the proteins were visualized in separated fluorescence and luminescence channels. For fluorescence detection, Alexa-647 and Cy-3 conjugated antibodies were used. Chemiluminescence was detected with HRP conjugated antibodies using Pierce ECL Western blotting substrate (32106, ThermoFisher). Multiple proteins were detected on the same membrane using the ChemiDoc XRS + Imaging System (Biorad), and labelling intensity was normalized to the stain-free signal. The molecular weights of the proteins were verified using a prestained protein ladder (ab116028, Abcam). Data were analyzed by densitometry in ImageJ using the gel quantification plugin.

### Synapse density and synaptic GABA receptor quantifications

The density of glutamatergic and GABAergic synapses was quantified using a previously established method [23]. For synapse analysis in vitro, five 66.5 × 66.5x5 µm ROIs per condition per experiment were selected at random positions, containing the soma and proximal dendrites of single neurons. In brain sections, five 51 × 51x10 µm ROIs per condition per experiment were selected in the left and right cerebral cortex layers 3–5. The confocal stacks were obtained using the LSM710 confocal microscope (100 × alpha Plan-Apochromat objective, NA 1.46; Carl Zeiss, Jena, Germany). Structurally complete synapses were identified by the overlapping immunolabelling of pre- and postsynaptic markers and quantified with the Synapse Counter plugin for ImageJ (https://github.com/SynPuCo/SynapseCounter). Our synapse quantification approach detects the majority of inputs, which a particular cell receives from its local network partners. The expression of pre- and postsynaptic GABA_B_ and GABA_A_ receptors was evaluated by immunofluorescence intensity analysis. Pre- and postsynaptic structures were analyzed using Synapse Counter, and mean pixel intensities were determined as estimates of protein expression changes.

### STED microscopy of postsynaptic scaffolds

The morphology of postsynaptic scaffolds was investigated by STED microscopy using a previously established method [23]. We employed the time-gated Leica TCS SP8 microscope (Wetzlar, Germany), which is equipped with a white light pulse laser (WLL2) and gated hybrid detection. An oil immersion HCX PL APO STED 100x (numerical aperture 1.4) objective was used. The ultrastructure of postsynaptic scaffolds within structurally complete synapses was analyzed. First, 23.8 × 23.8 µm single-plane confocal images were obtained using 488 and 633 nm excitation wavelengths for the post- and presynaptic markers, respectively. Then, the STED scans were obtained in the same stage position using 488 nm excitation and 592 nm depletion lasers. The detection time-gating interval was set to 6–10 ps post-pulse time window. All settings were the same throughout the experiments. The raw data were deconvolved using Hyugen's software. The binary masks of single synaptic scaffolds were generated using automated thresholding (Otsu method) in ImageJ, and the mask area was measured.

### Whole-cell patch-clamp recordings

Action potential generation and spontaneous postsynaptic currents were measured in mature neuronal cultures by the whole-cell patch-clamp method using Axopatch 200B amplifier (Molecular Devices, San Jose, CA, U.S.A.) and analyzed with pClamp software 10.6 (Molecular Devices). Microelectrodes of 1.5 mm thin-walled filamented borosilicate glass (World Precision Instruments, Friedberg, Germany) were pulled with a DMZ-Universal Puller (Zeitz Instruments, Martinsried, Germany) and polished to a final resistance of 3–4 MΩ. Neurons were voltage-clamped at -60 mV, the signals were filtered at 1.0 kHz and recorded with 10 kHz. Series resistance and cell capacitance were compensated prior to the recordings. BrainPhys basal medium (05790, STEMCELL Technologies) was used as an extracellular solution. The pipette solution contained 140 mM KCl, 1 mM CaCl_2_·2H_2_O, 4 mM MgCl_2_, 10 mM HEPES, 0.4 mM Na_2_-GTP, 4 mM Mg-ATP and 10 mM EGTA (pH 7.3). Action potential thresholds were determined by applying incrementally increasing depolarizing current steps of 500 ms duration. Passive membrane properties and firing patterns were characterized to discriminate the fast-spiking inhibitory interneurons (spiking frequency ≥ 5 Hz) and the excitatory neurons (spiking frequency below 5 Hz). For the recording miniature inhibitory postsynaptic currents (mIPSCs) we applied 1 µM tetrodotoxin (TTX, Tocris, Bristol, UK) to prevent action potential-driven synaptic release. The inhibitory postsynaptic currents were pharmacologically isolated using 10 µM glutamate receptor antagonist DNQX (Tocris) and 10 µM NMDA receptor antagonist D-APV (Tocris).

### Spontaneous network activity recordings

Spontaneous activity in neuronal networks was measured using cell culture compatible square 8 × 8 electrode MEA (60MEA200/30iR-Ti, Multi Channel Systems, Reutlingen, Germany), on which primary neurons were grown. To evaluate the effects of ECM depletion, we recorded the baseline activity prior to the application of digesting enzymes and compared it with the post-treatment activity after 16 h. The control cultures were treated with 0.1 M PBS. To study the impact of ECM depletion on GABAergic neurotransmission, GABA_A_ (6 µM bicuculline metiodide, Tocris) or GABA_B_ (100 µM CGP46381, Tocris) receptor antagonists were applied. After 30 min of incubation with the antagonist, network activity was recorded. The effects of GABA antagonists were evaluated with reference to neuronal activity after ECM depletion or control treatment. In all experiments, we recorded spontaneous network activity for 15 min (temperature stabilized at 35 °C, gas exchange prevented) using the MEA2100 60-channel headstage with the sampling frequency of 40,000 Hz using MC Rack. For each electrode, the mean firing rate (MFR) and mean bursting rate (MBR) were analyzed in MatLab with the SpyCode toolbox generously provided by Dr. Michela Chiappalone [9].

### Network activity simulations

To evaluate the impact of connectivity alterations versus the synaptic strength changes induced by ECM depletion, we implemented a previously established computational approach [21]. Experimentally observed effects of ECM depletion were reconstructed using an in silico network of spiking neurons. The key variables of our model define neuronal response properties and network connectivity. The physiology of fast-spiking interneurons and primary excitatory neurons was replicated using previously defined parameters [41]. Network circuitry was defined by the sparseness of connectivity (C_exc_, C_inh_) and synaptic weights (W_exc_, W_inh_). The sparseness of connectivity was defined as the proportion of all neurons of a certain type providing the input to a single cell on average: C_exc_ = 0.25*N_exc_ for excitatory, C_inh_ = 0.15*N_inh_ for inhibitory input in the control condition. Synaptic weights were set as absolute values of postsynaptic membrane potential changes after activation of a synapse. We modified C_inh_ and W_inh_ within a range of biologically feasible values to determine their impact on neuronal network activity. For each simulation instance, network connectivity was newly generated, mimicking the intrinsic variability of real neuronal networks.

### Statistics

For non-normally-distributed datasets, data were evaluated by Kruskal–Wallis tests using OriginPro2020 software. For multiple comparisons, Bonferroni correction was applied. Data were presented as box plots depicting the medians (lines inside boxes)/ means (filled squares inside boxes) ± IQR (boxes) with 10% and 90% ranks as whiskers. For normally distributed datasets, e.g. the in silico simulations, data were evaluated by two-tailed independent Student’s t-tests. Data were shown as mean ± s.e.m. columns. Data points were indicated as diamonds. With the significance level α set to 0.05, the *p* values < 0.05 were defined to indicate statistically significant differences.

## Results

### ECM depletion reduces inhibitory synapse density in vitro

**Fig. 1:**
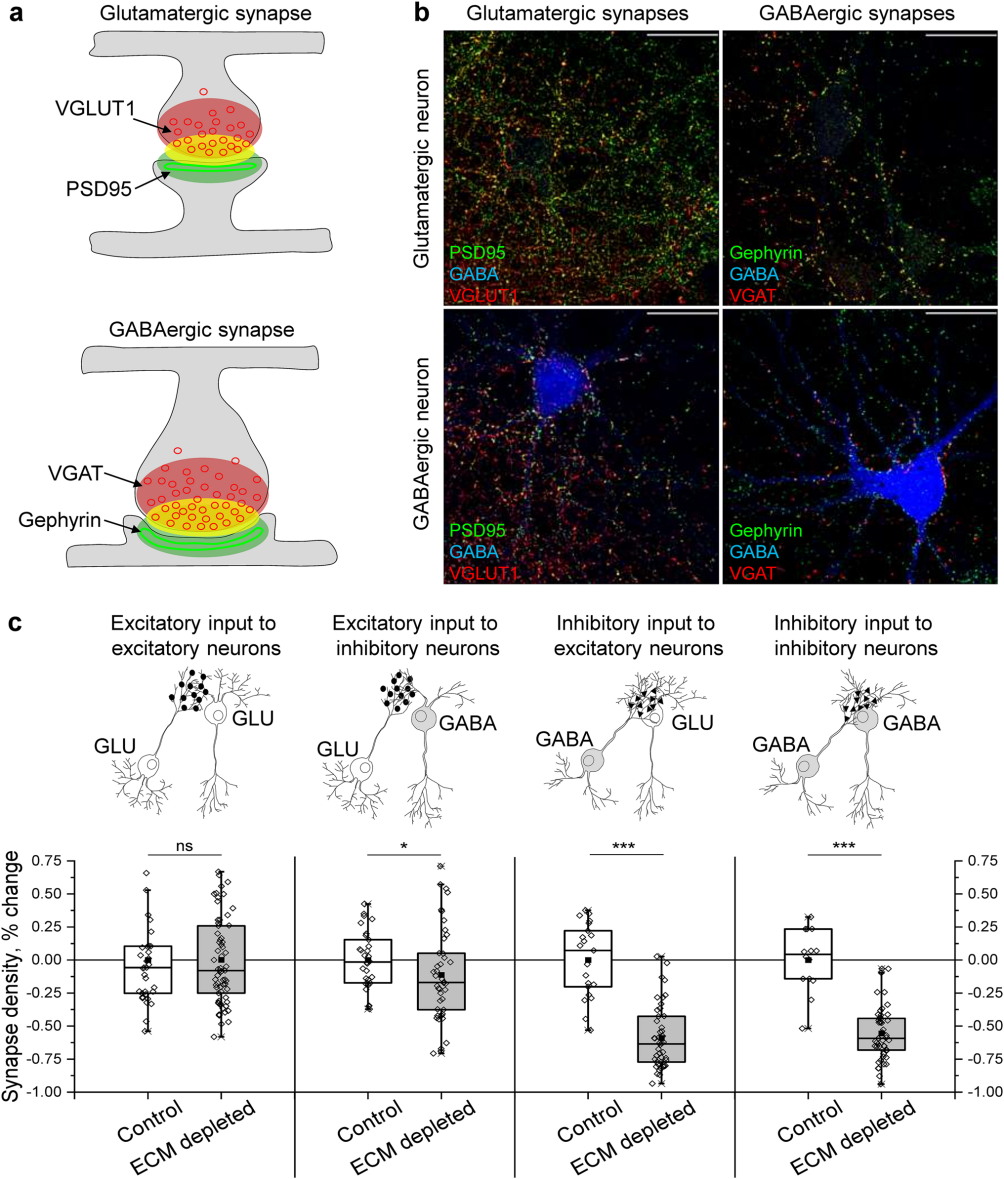
Excitatory and inhibitory synapse densities decrease after ECM depletion in vitro***.*** **a** Overlapping immunolabelling of presynaptic (red) and postsynaptic (green) markers was used to detect structurally complete synapses (yellow). **b** The density of glutamatergic (PSD95-VGLUT1) and GABAergic (gephyrin-VGAT) synapses was measured with reference to GABA immunoreactivity. Representative micrographs are shown. Scale bars, 30 µm. **c** Synapse density changes were calculated as differences with mean values of corresponding control experiments. Data are shown for each neuron examined (*n* ≥ 20 neurons per condition, results obtained from 5 independent experiments). *GLU* glutamate. Data are medians (lines inside boxes)/ means (filled squares inside boxes) ± IQR (boxes) with 10/ 90% ranks as whiskers. Open diamonds are data points. The asterisks indicate significant differences with control, based on Kruskal–Wallis tests (***p < 0.001, **p < 0.01). *ns* not significant

Measuring synapse density changes provides an indirect but straightforward estimate of neuronal network connectivity alterations [21]. Thus, we first quantified the density of structurally complete synapses in mature networks of primary murine neurons. The networks consisted of ~ 67% principal excitatory neurons and ~ 33% inhibitory interneurons (which is slightly higher than 20–25% in vivo [38, 66]) and were fully mature after 21 days of cultivation, indicated by the appearance of PNNs, which are condensed ECM layers, in neuronal cultures (Figs. S1 and S2). The density of both GABAergic and glutamatergic synapses was quantified using GABA as a marker of inhibitory perikarya, allowing for the gross estimation of network connectivity. The co-labelling of vesicular glutamate transporter type 1 (VGLUT1) and postsynaptic density protein 95 (PSD95) indicated the excitatory inputs to excitatory and inhibitory neurons, while the co-labelling of vesicular GABA transporter (VGAT) and gephyrin signified inhibitory inputs to excitatory and inhibitory neurons (Fig. 1a, b).

The density of glutamatergic and GABAergic synapses was differently affected by enzymatic ECM depletion (500 mU/ml chondroitinase ABC [ChABC], 16 h). Compared with the control, ECM depletion reduced excitatory input to inhibitory neurons by 11.2 ± 5.5%, inhibitory input to excitatory neurons by 60.3 ± 5.7% and inhibitory input to inhibitory neurons by 53.7 ± 5.9% (Fig. 1c). ECM depletion did not affect neuronal survival (Fig. S5). Because GABAergic synapses were affected more strongly than glutamatergic ones, we concluded that ECM depletion preferentially reduced inhibitory connectivity in vitro.

### ECM depletion reduces inhibitory synapse density in vivo

**Fig. 2:**
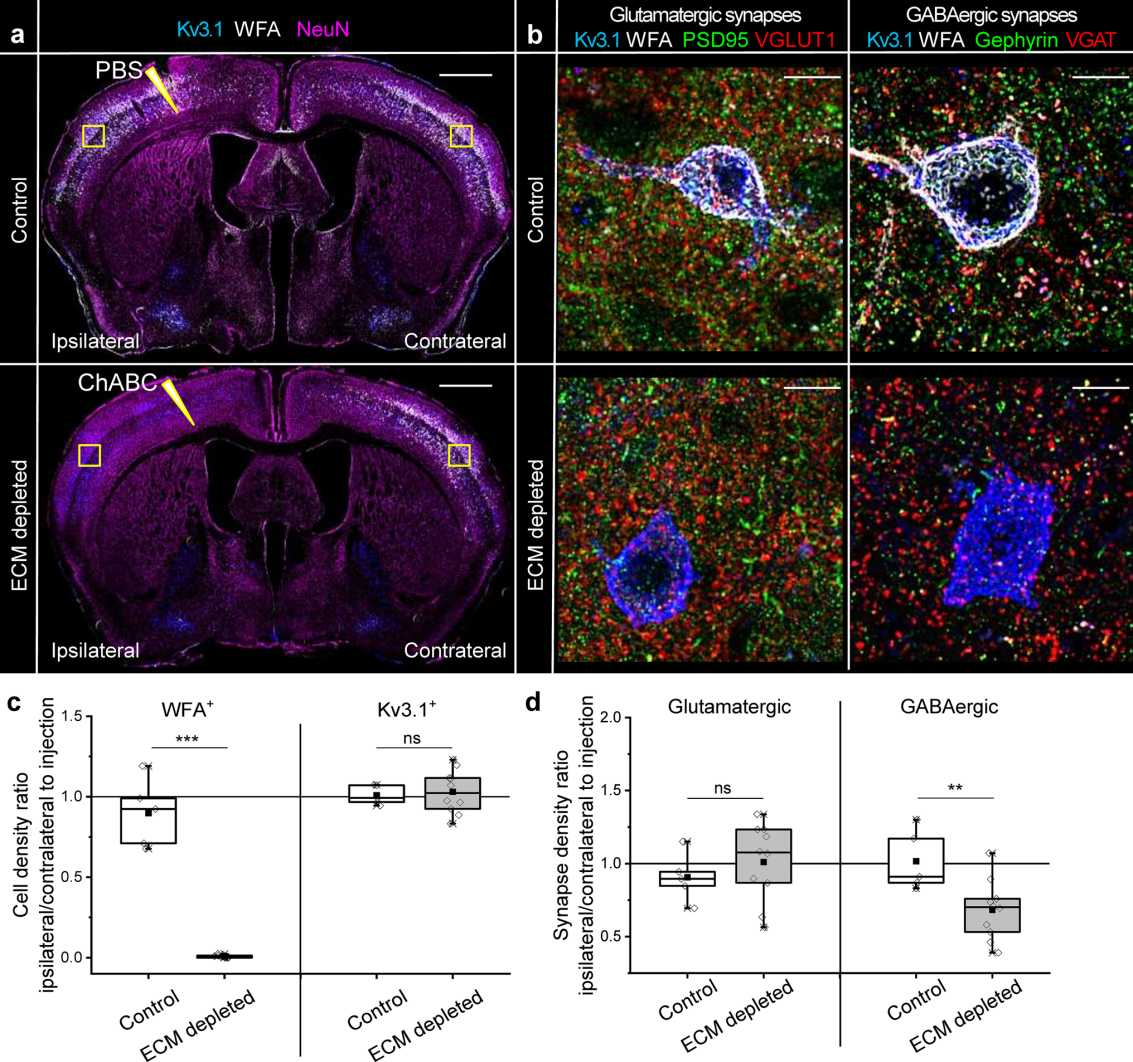
Inhibitory synapse density decreases after ECM depletion in vivo*.* **a** Neuronal nuclei (NeuN, magenta), fast spiking interneurons (Kv3.1, blue) and PNNs (WFA, *Wisteria floribunda* agglutinin, white) were immunohistochemically labeled in brain sections obtained from mice treated with chondroitinase ABC (ChABC, ECM depleted) or phosphate buffered saline (PBS, control) for 16 h. Sharp triangles indicate intracortical injection sites. Squares indicate the regions in which cell and synapse densities were analyzed. Scale bar, 1 mm. **b** The density of glutamatergic (PSD95-VGLUT1) and GABAergic (gephyrin-VGAT) synapses was measured in somatosensory cortex layers 3–5. Maximum projections of 56.7 × 56.7x5 μm regions ipsilateral to the injection sites are shown. Scale bars, 10 µm. **c** Changes in PNN^+^ and Kv3.1^+^ neuron densities were quantified as ipsilateral to contralateral ratios. **d** Changes in glutamatergic and GABAergic synapse densities were calculated as ipsilateral to contralateral ratios. Data are shown for each animal examined (*n* ≥ 5 animals per condition). Data are medians (lines inside boxes)/ means (filled squares inside boxes) ± interquartile ranges (IQR; boxes) with 10/ 90% ranks as whiskers. Open diamonds are data points. The asterisks indicate significant differences with control, based on Kruskal–Wallis tests (***p < 0.001, **p < 0.01). *ns* not significant

Further, we analyzed the density of glutamatergic and GABAergic synapses in somatosensory cortex layers 3–5 following ECM depletion in vivo. Intracortical injection of ChABC (500 mU in 3 μl 0.1 M phosphate-buffered saline [PBS], 16 h) unilaterally depleted ECM in the brain, indicated by the absence of PNNs, which in the brain are found around the fast-spiking interneurons expressing a specific potassium channel Kv3.1. The procedure did not affect the density of Kv3.1^+^ neurons and did not alter PNN expression in the contralateral hemisphere (Fig. 2a, c). ECM depletion did not influence the density of glutamatergic synapses but significantly reduced GABAergic synapses in the cortex by 42 ± 6% (Fig. 2b, d). Hence, ECM depletion reduced inhibitory connectivity in vivo.

### ECM depletion alters electrophysiological properties of neurons

**Fig. 3:**
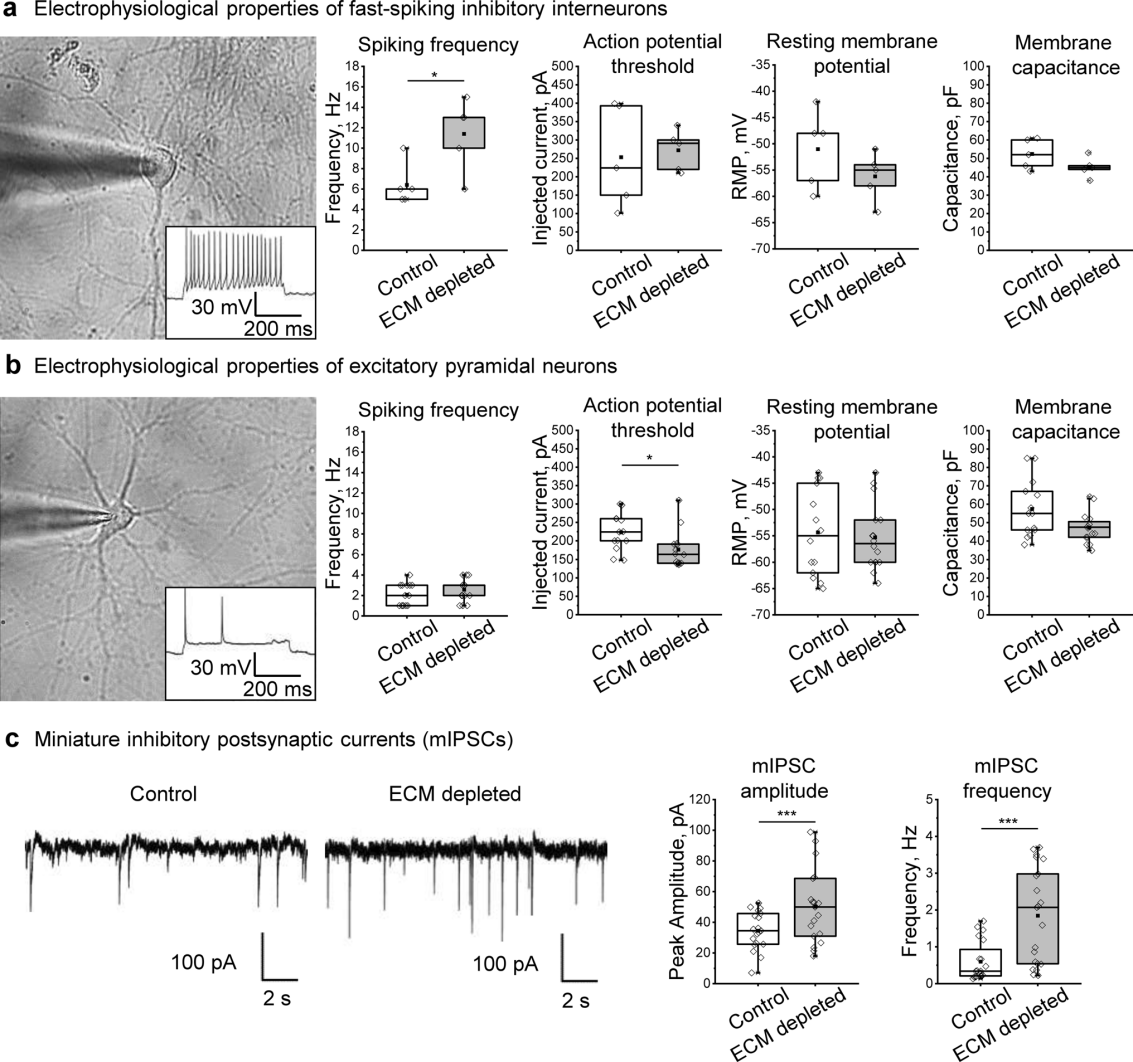
ECM depletion affects electrophysiological properties in single neurons and strengthens inhibitory synapses. Spiking frequency, action potential threshold, resting membrane potential, and membrane capacitance were evaluated in the fast-spiking inhibitory interneurons (**a**) and the excitatory neurons (**b**). The results were obtained from 5 independent experiments. **c** Patch clamp recordings in presence of sodium channel blocker (TTX) and glutamate receptor antagonists (DNQX and D-AP5) reveal miniature inhibitory postsynaptic currents (mIPSCs). Representative current tracks exemplify mIPSCs detected in control and ECM depleted cultures. Quantifications of mIPSC amplitude and frequency indicate that ECM depletion increased the total inhibitory input to single neurons (*n* ≥ 19 neurons per condition, results obtained from 5 independent experiments). Data are medians (lines inside boxes)/ means (filled squares inside boxes) ± IQR (boxes) with 10/ 90% ranks as whiskers. Open diamonds are data points. The asterisks indicate significant differences with control, based on Kruskal–Wallis tests (*p < 0.05, ***p < 0.001)

To investigate whether the reduced inhibitory connectivity after ECM depletion is accompanied by neuronal electrophysiology changes, we analyzed passive membrane properties (resting membrane potential and capacitance) and action potential generation (threshold, frequency) using somatic patch-clamp recordings (Fig. 3a, b). We applied incrementally increasing depolarizing current steps of 500 ms duration to determine action potential thresholds in excitatory and inhibitory neurons. Neuronal types were discriminated against by their spiking behavior and resting membrane potentials. Like in previous studies [46, 57, 68], the inhibitory interneurons exhibited considerably faster spiking dynamics (spiking frequency ≥ 5 Hz) than the excitatory neurons (spiking frequency below 5 Hz). ECM depletion significantly increased the spiking frequency of inhibitory interneurons but did not alter action potential threshold, resting membrane potential, and capacitance (Fig. 3a). In excitatory neurons, ECM depletion reduced the action potential threshold, rendering these cells more excitable (Fig. 3b). Spiking frequency and passive membrane properties were not altered in excitatory neurons.

### ECM depletion increases the strength of inhibitory synapses

Since ECM depletion preferentially reduced the density of inhibitory synapses, we further asked whether the strength of inhibitory input to individual neurons is functionally altered. Spontaneous neurotransmitter release in inhibitory synapses was measured by recording miniature inhibitory postsynaptic currents (mIPSCs) in mature neurons (Fig. 3c).

For recording mIPSCs, 1 µM tetrodotoxin (TTX) was applied to prevent action potential-driven synaptic release, and a mixture of glutamate receptor antagonists (10 µM DNQX and 10 µM D-APV) was added to isolate inhibitory currents. Interestingly, ECM depletion increased both amplitude and frequency (Fig. 3c, d) of mIPSCs. While the higher mIPSC amplitude indicates elevated neurotransmitter content per synaptic vesicle [27] or increased number and conductance of postsynaptic receptors [49], the higher frequency can result from increased release probability and the number of synapses [58]. Considering the reduced synapse number (Fig. 1c), these data show that ECM depletion increased the strength of inhibitory synapses. We suggest that this effect reflects a homeostatic mechanism for restoring the excitation-inhibition (E-I) balance in case of losing a portion of inhibitory synapses.

### ECM depletion reduces presynaptic expression of GABA_B_ receptors in inhibitory synapses

**Fig. 4:**
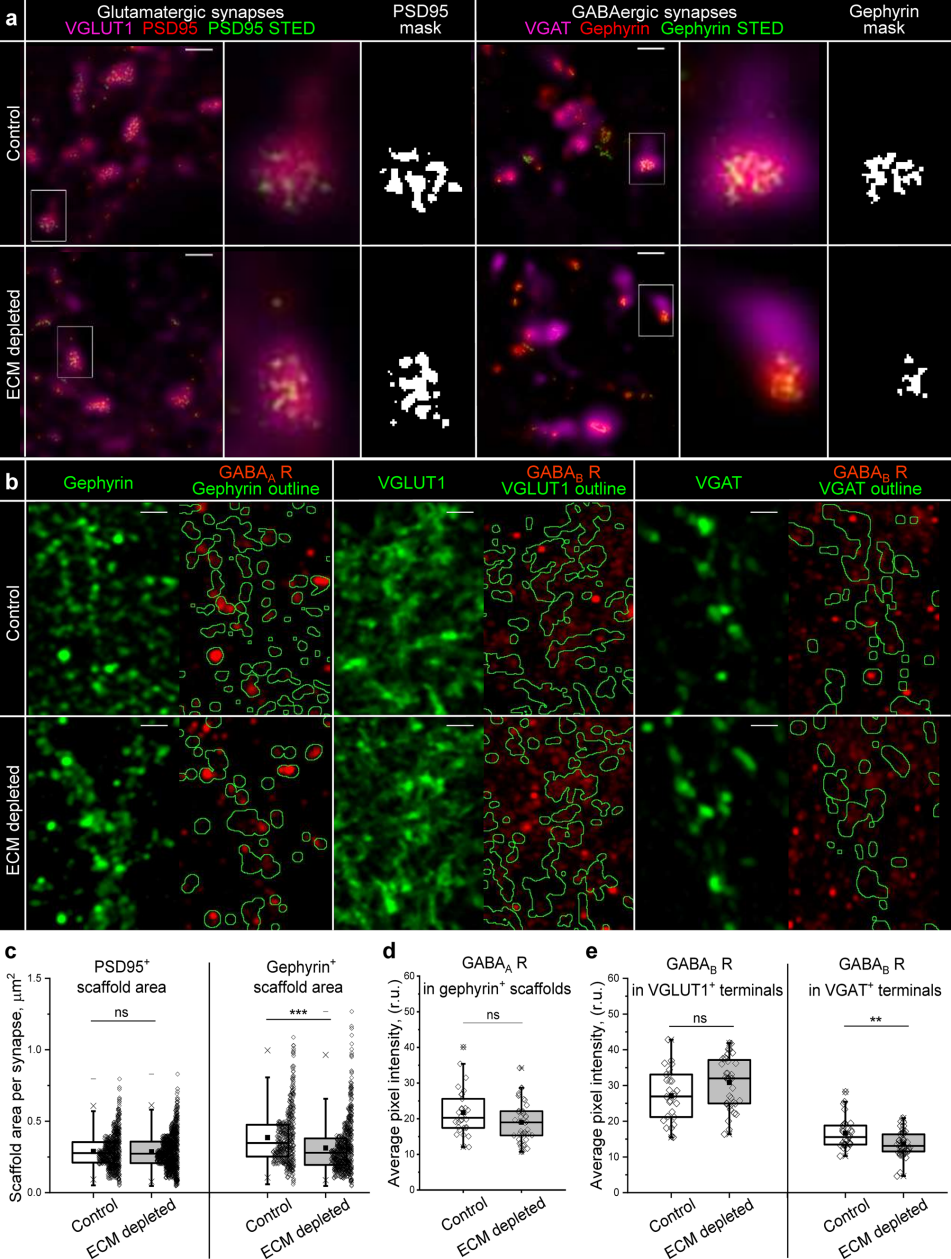
ECM depletion alters the pre- and postsynaptic organization of inhibitory synapses. **a** Stimulated emission depletion (STED) microscopy resolves the morphology of presynaptic scaffolds in glutamatergic and GABAergic synapses. Scale bars, 1 μm. Single synapses highlighted with white rectangles are magnified and the corresponding masks of postsynaptic scaffolds are shown. **b** The panel illustrates the analysis of GABA_A_ receptor (GABA_A_ R) expression in inhibitory postsynapses (gephyrin^+^ areas), GABA_B_ receptor (GABA_B_ R) expression in excitatory (VGLUT1^+^ areas) and inhibitory (VGAT^+^ areas) postsynapses. The outlined areas (green) depict the regions in which the immunoreactivity of GABA receptors was measured. Scale bars, 2 μm. **c** The area of scaffolds containing PSD95 or gephyrin was quantified in single synapses (n ≥ 580 synapses per condition, results from 5 independent experiments). **d** Immunoreactivity of GABA_A_ receptors in GABAergic postsynapses. **e** Immunoreactivity of GABA_B_ receptors in glutamatergic and GABAergic presynapses. **d**, **e** The average pixel intensity was quantified for each neuron examined (n ≥ 30 cells per condition, results from 5 independent experiments). Data are medians (lines inside boxes)/ means (filled squares inside boxes) ± IQR (boxes) with 10/ 90% ranks as whiskers. Open diamonds are data points. The asterisks indicate significant differences with control, based on Kruskal–Wallis tests (***p < 0.001, **p < 0.01). ns, not significant

To understand how ECM depletion facilitates inhibition, we examined the ultrastructural organization of inhibitory and excitatory postsynapses and analyzed the distribution of GABA receptors. We used stimulated emission depletion (STED) microscopy to uncover the morphology of gephyrin and PSD95 containing scaffolds within structurally complete GABAergic and glutamatergic synapses (Fig. 4a).

By analyzing the area of binary masks representing single synaptic scaffolds, we determined that ECM depletion reduced the size of gephyrin, but not PSD95 scaffolds (Fig. 4c). Because gephyrin scaffolds are essential for the clusterization of postsynaptic GABA_A_ receptors, we measured the immunoreactivity of GABA_A_ receptors inside gephyrin containing postsynapses (Fig. 4b, d), but found no significant changes after ECM depletion. The total expression of GABA_A_ receptors was not affected (Fig. S6).

GABA_B_ receptors act as negative regulators of neurotransmitter release on the presynaptic side of both excitatory and inhibitory synapses. By measuring the immunoreactivity of GABA_B_ receptors on VGLUT1^+^ and VGAT^+^ presynaptic terminals, we revealed that ECM depletion preferentially reduces GABA_B_ receptor expression in GABAergic presynapses (Fig. 4b, e). These results suggest that the increased strength of inhibitory synapses after ECM depletion is associated with reduced reciprocal inhibition of neurotransmitter release. Neither total nor postsynaptic expression of GABA_B_ receptors was significantly influenced by ECM depletion (Figs. S6 and S7).

### ECM depletion increases neuronal activity and facilitates spiking-bursting transitions

**Fig. 5:**
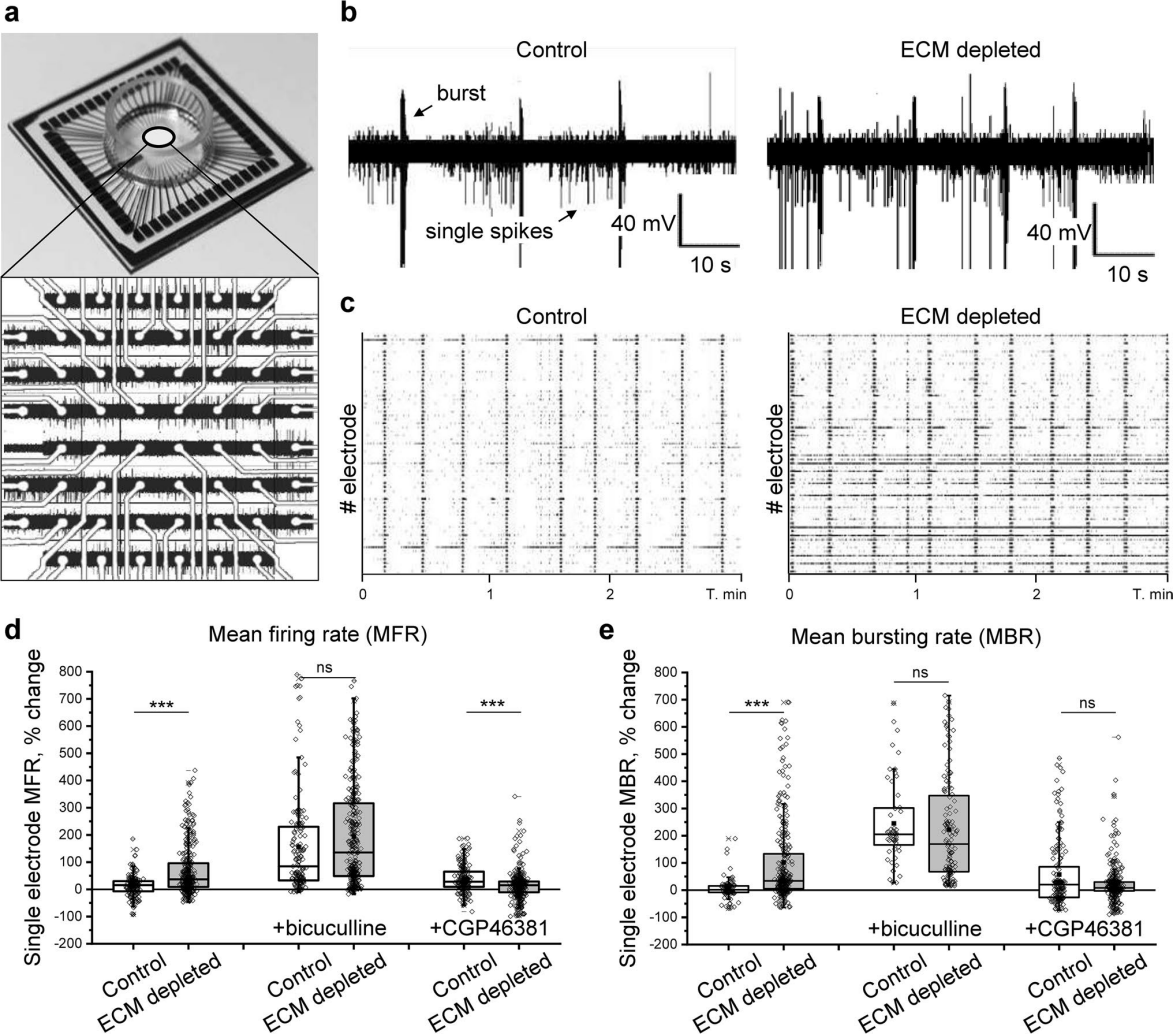
The increase of neuronal network activity after ECM depletion is inhibition dependent. **a** Neuronal network activity was examined using multiple electrode arrays (MEAs). The panel demonstrates the layout and network activity recorded on a MEA chip with a square array of 59 electrodes. **b** Representative voltage tracks exemplify spikes and bursts detected by single electrodes in control and ECM depleted cultures. **c** Raster plots show synchronized network activity in control and ECM depleted cultures. Black ticks are single spikes, magenta bars are burst events. The changes of (**d**) mean firing rate (MFR) and (**e**) mean bursting rate (MBR) were quantified for single electrodes as the differences with the baseline activity of the same electrode before treatment (*n* ≥ 169 electrodes per condition, results from 5 independent experiments). The effects of GABA_A_ and GABA_B_ receptor blockage were analyzed by comparing single electrode activity before and after incubation with the antagonist (6 μM bicuculline and 100 μM CGP46381, respectively). Data are medians (lines inside boxes)/ means (filled squares inside boxes) ± IQR (boxes) with 10/ 90% ranks as whiskers. Open diamonds are data points. The asterisks indicate significant differences with control, based on Kruskal–Wallis tests (***p < 0.001). *ns* not significant

Knowing that ECM depletion reduces inhibitory connectivity, increases the strength of both excitatory and inhibitory synapses, elevates the firing frequency of fast-spiking inhibitory interneurons, and enhances the excitability of slower excitatory neurons, we asked how these opposing changes affect neuronal activity at the network level. Spontaneous network activity was investigated using multiple electrode arrays (MEAs). Within this methodology, neuronal cultures are grown on an array of electrodes, each detecting population spikes and bursts, generated by small groups of neurons (10–15 in our experiments), located within 100 µm radius from the center of the electrode (Fig. 5a, b). While the frequency of spikes measured as the electrode mean firing rate (MFR) represents general neuronal activity, the network phase transitions are represented by bursting behavior, which was evaluated as mean bursting rate (MBR) changes. In mature networks, spiking-bursting transitions are mostly synchronized, as indicated by the alignment of burst events (Fig. 5c). Therefore, MBR measurements also partially reflect neuronal network synchrony.

We analyzed how ECM depletion alters neuronal activity and bursting behavior by measuring MFR and MBR changes after ChABC (500 mU/ml, 16 h) or control (PBS, 16 h) treatment. The activity recorded by single electrodes was compared to the baseline activity of the same electrode before treatment (Fig. 5d, e). On average, ECM depletion increased MFR by 69.8 ± 5.2% (mean ± s.e.m.) and MBR by 102.0 ± 9.6% (mean ± s.e.m.). The increase of neuronal network activity and facilitated bursting was inhibition-dependent since the blockage of GABA_A_ receptors (6 µM bicuculline metiodide for 30 min) levelled the effect of ECM depletion. The blockage of GABA_B_ receptors (100 µM CGP46381 for 30 min) moderately enhanced neuronal activity. After ECM depletion, the effect of GABA_B_ receptor blockage was significantly reduced, as indicated by MFR quantification after treatment with CGP46381. Hence, the increase of neuronal network activity after ECM depletion functionally involved the downregulation of GABA_B_ receptors.

### Loss of inhibitory synapses after ECM depletion renders inhibitory control inefficient

**Fig. 6:**
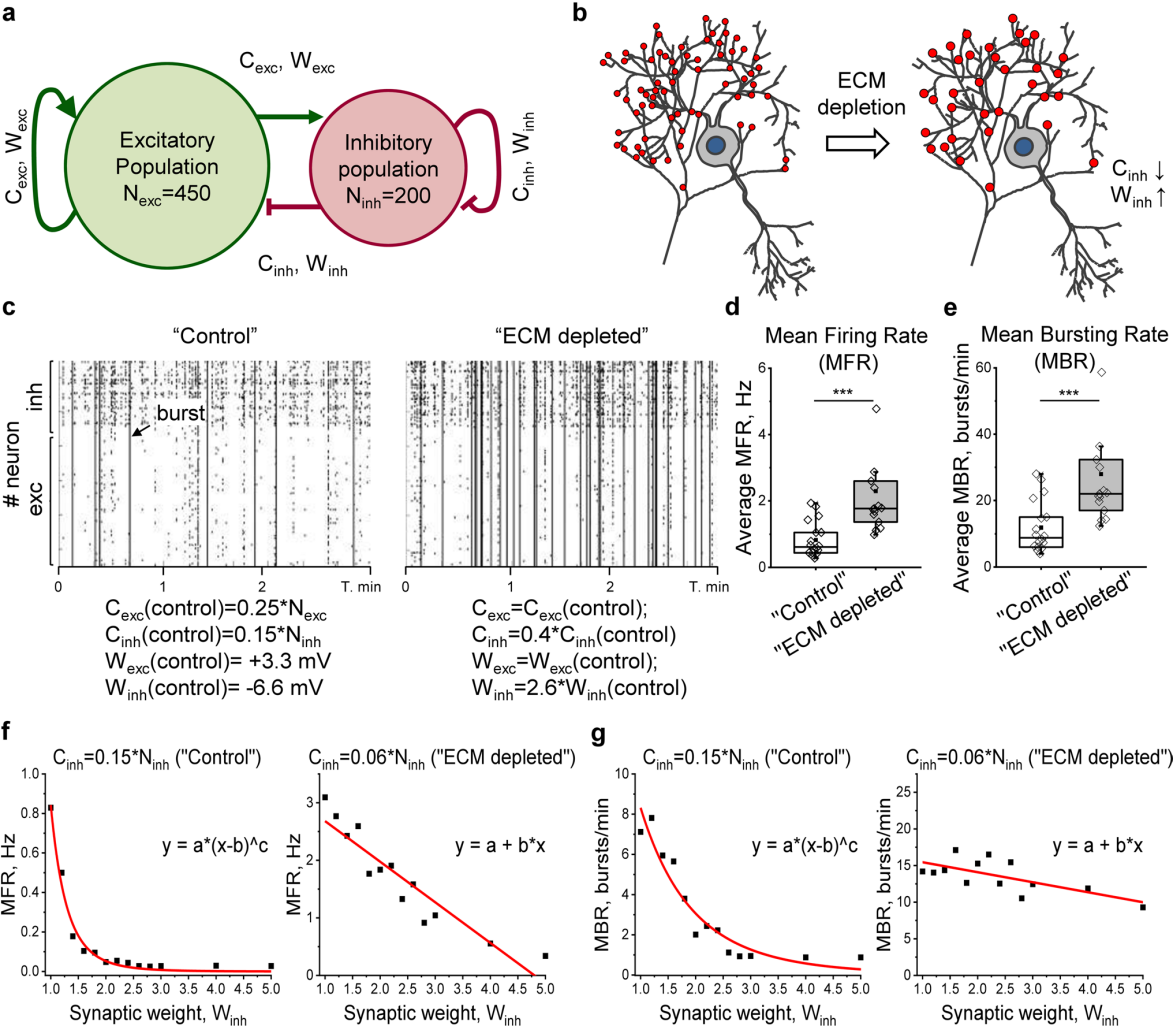
Network activity simulation in silico indicates the prevailing role of inhibitory connectivity reduction following ECM depletion. **a** The schematic drawing illustrates the model of the spiking neuron network. N_inh_ and N_exc_ are numbers of inhibitory and excitatory neurons, W_inh_ and W_exc_ are weights of corresponding synapses. **b** The ECM depletion was mimicked by tuning C_inh_ and W_inh_ parameters in accordance with experimentally observed changes. **c** Raster plots exemplify the activity of “control” and “ECM depleted” networks. The corresponding simulation parameters are depicted. Ticks are single spikes, vertical dashes indicate burst events. The quantification of network average (**d**) mean firing rate (MFR) and (**e**) mean bursting rate (MBR) is shown for “control” and “ECM depleted” simulation conditions. Data are medians (lines inside boxes)/ means (filled squares inside boxes) ± IQR (boxes) with 10/ 90% ranks as whiskers. Open diamonds are data points. The asterisks indicate significant differences with the control, based on Kruskal–Wallis tests (***p < 0.001). (**f**) MFR and (**g**) MBR are quantified in a range of W_inh_ changes for “control” and “ECM depleted” inhibitory connectivity. Note that the reduction of inhibitory connectivity switches the dependence of network activity on inhibitory synapse weight from power law to linearity. Squares indicate the mean of simulation repetitions, fit functions are shown in red. For each condition, 15 independent simulation experiments were performed

The number and strength of GABAergic synapses are essential determinants of inhibitory input that control the activity and synchronization in neuronal networks. We revealed that these parameters undergo opposing changes after ECM depletion, implying the necessity to understand their interaction at the network level. Using the earlier elaborated method [21], we reconstructed the observed alterations in silico (Fig. 6a). Excitatory and inhibitory connectivity was defined as the proportion of all neurons providing the input to a single cell (C_exc_ = 0.25*N_exc_, C_inh_ = 0.15*N_inh_ in “control”), based on the previous study [41] and connectivity estimations in neuronal cultures [51]. The strength of single connections was defined by synaptic weights reflecting membrane potential changes upon synapse activation (W_exc_ =  + 3.3 mV, W_inh_ = -6.6 mV in “control”). Thereby, excitation and inhibition were balanced, and the computations were performed in a near-critical state (Fig. S8) characterized by stable spiking-bursting transitions. ECM depletion was simulated by modifying inhibitory connectivity (C_inh_) and synaptic weights (W_inh_) in accordance with experimentally observed changes (Fig. 6b). In agreement with in vitro experiments, ECM depletion increased network MFR (Fig. 6c, d) and bursting rates (Fig. 6c, e). Of note, our approach closely resembled the intrinsic variability in real neuronal networks, because the connectivity matrix was newly generated for each simulation instance.

To compare the impact of C_inh_ and W_inh_ on the resulting neuronal activity, we measured MFR and MBR over a range of different C_inh_ and W_inh_ values (Fig. S9). Excitatory input parameters C_exc_ and W_exc_ were set to control values. The reduction of C_inh_ negatively and linearly correlated with network MFR (*r* =  – 0.98; *p* < 0.01) and MBR (*r* =  – 0.92; *p* < 0.01), while the increased C_inh_ strongly diminished neuronal activity. Under moderate decrease of C_inh_ (C_inh_ = 0.125*N_inh_ and C_inh_ = 0.1*N_inh_), increasing W_inh_ partially compensated MFR and MBR changes. However, when C_inh_ was set in accordance with ECM depletion effect in vitro (40% of control, C_inh_ = 0.06*N_inh_), both MFR and MBR remained elevated in a broad range of W_inh_. While changing inhibitory connectivity from C_inh_ = 0.15*N_inh_ in “control” simulation to C_inh_ = 0.06*N_inh_ in “ECM depleted” simulation, the dependence of MFR and MBR on W_inh_ switched from a power law to linearity (Fig. 6f, g). Conclusively, the reduction of inhibitory connectivity after ECM depletion rendered inhibitory control inefficient and increased the resulting activity of neuronal networks.

## Discussion

Here we demonstrate that the brain ECM supports the maintenance of neuronal network E-I balance by retaining inhibitory connectivity. ECM depletion preferentially decreases the density of inhibitory synapses and the size of inhibitory postsynaptic scaffolds, while it homeostatically increases inhibitory synapse strength. Commonly, inhibitory synapse scaling downregulates neuronal network activity and synchronization, providing a key mechanism for neuronal activity adjustment [65]. After ECM depletion, the degree of inhibitory connectivity reduces to an extent that inhibitory synapse scaling is no longer efficient in controlling the state of neuronal networks. As a result, neuronal network activity and synchrony increase.

We observed that ~ 60% of GABAergic synapses are lost already 16 h after ECM depletion. How could such a major decrease in synapse density occur without increased neuronal cell death? Recent findings indicate that neuronal networks are continuously remodelled and that synapses dynamically wane and re-emerge within a few days [52]. Inhibitory synapses are especially dynamic, about 60% of them retract and return at the same place within 4 days [71]. Intuitively, this structural volatility but spatial persistency implies the existence of a stable framework to secure the integrity of neuronal circuits. Our data suggest that the brain ECM provides such a framework to stabilize inhibitory connectivity. After ECM depletion, the reduction of GABAergic synapse density may be due to the increased volatility and inability of synaptic boutons to find their postsynaptic site. The demarcation of postsynaptic sites has been proposed to be defined by PNNs [26, 63], the facet-like ECM coatings that compartmentalize neuronal surface. In mice lacking several ECM glycoproteins, the defective formation of PNNs associated with an increased ratio of excitatory to inhibitory synapses [34]. PNNs covering parvalbumin-containing interneurons maintain orthodenticle homeobox 2 (Otx2) dependent breaks on adult brain plasticity [2, 5]. We hypothesize that a less dense ECM layer may play a similar role on neurons devoid of PNNs.

At the level of single synapses, ECM depletion increases inhibitory synapse strength, as indicated by the increased mIPSC amplitude and frequency together with the decreased synapse density. With STED microscopy, we observed the reduced size of postsynaptic gephyrin scaffolds after ECM depletion. However, despite the known role of gephyrin for postsynaptic GABA_A_ receptor clustering and stabilization [15], neither expression nor localization of GABA_A_ receptors was affected. Apparently, the gephyrin-containing scaffolds condensed and retained GABA_A_ receptors. On the presynaptic side, we found a significant reduction of GABA_B_ receptor expression on VGAT^+^ terminals, which functionally associated with the diminished sensitivity to the GABA_B_ antagonist CGP46381. Presynaptic GABA_B_ receptors act as activity-dependent regulators of neurotransmitter release. Upon repetitive stimulation, the spillover of GABA activates presynaptic GABA_B_ receptors, transiently reducing subsequent neurotransmitter release [18, 53]. Our results indicate that ECM depletion attenuated this mechanism and increased inhibitory synapse strength via presynaptic facilitation. It remains unclear how exactly the ECM regulates GABA_B_ receptor localization and function. The extracellular complement control protein module CCP1 of GABA_B_ R1 subunit interacts with laminin α5 subunit and fibulin-2 of the ECM [8, 55], but the functional consequences of this interaction need further investigation.

Our data shows that the increased synaptic strength does fully compensate for the reduction of inhibitory synapse number after ECM depletion. As a result, the reduction of inhibitory connectivity outweighs the increased inhibitory synapse strength, and the E-I balance switches towards excitation. Thereby, ECM depletion increases neuronal activity and network synchrony, as evidenced by MEA recordings and in silico simulations. A similar increase of network activity could arise from the reduced excitatory input in fast-spiking interneurons, as observed in the hippocampus after ECM depletion [37], and the decreased rate of their spontaneous firing, as observed in a model of peritumoral epilepsy [67]. In this work, we observed neither significant changes of excitability [37] nor increased excitatory input [24] in fast-spiking interneurons, which were previously described in a similar model of ECM depletion. Unlike the VGLUT2^+^ thalamic inputs in the visual cortex [24, 48], ECM depletion does not significantly alter the local excitatory connectivity in somatosensory cortex layers 3–5, as indicated by the VGLUT1^+^ synapse quantifications. Although the possibility of increased stimulation of GABAergic interneurons via the long-range thalamocortical projections remains, our results indicate that inhibitory synapse stabilization is a key mechanism by which ECM supports E-I balance in local neuronal networks. In addition, E-I balance changes after ECM depletion may be further amplified by the increased excitability of excitatory neurons that we observed. However, the reduction of action potential threshold in excitatory neurons can be counterbalanced by the increased firing rate of inhibitory interneurons, which we also detected experimentally. The mechanism by which ECM depletion modifies electrophysiological properties of individual neurons potentially involves altered buffering of membrane-associated calcium [70], but remains to be further explored.

Altered E-I balance following the ECM breakdown is likely a key component of the pathophysiology of psychosis [50, 64] and epilepsy [1, 67]. In ischemic stroke, adjusting the E-I balance after ECM decomposition may, on the contrary, support neurological recovery. Post-stroke neuroplasticity is impaired by decreased neuronal excitability in perilesional brain areas [17, 73]. In light of the new evidence we present here, the transient decline in cortical ECM integrity after ischemia [22] may support neuronal network rewiring by stimulating neuronal activity. Hence, the controlled degradation of ECM could be a promising target in stroke therapy, as it may allow promoting neuronal activity and plasticity.

The therapeutic potential of ECM degradation in the injured brain will crucially depend on the precise targeting of ECM modifications. The crude ablation of the ECM elicits memory loss and learning deficits [40, 62]. In this study, we demonstrate that the near-complete ECM digestion disrupts criticality in neuronal networks, indicated by the switch of dependence between neuronal activity and inhibitory synapse strength from power law to linearity. The dynamic tuning of cortical circuits to criticality is essential for efficient information processing in the brain [30, 42, 45]. Refined tools for controlled ECM decomposition will not only expand opportunities for research but will also open new directions in neurorestorative therapies.

## Supplementary Information

Below is the link to the electronic supplementary material.

Supplementary file1 (TIF 76049 KB)

Supplementary file2 (TIF 59900 KB)

Supplementary file3 (TIF 148271 KB)

Supplementary file4 (TIF 125803 KB)

Supplementary file5 (TIF 34823 KB)

Supplementary file6 (TIF 43656 KB)

Supplementary file7 (TIF 23094 KB)

Supplementary file8 (TIF 2355 KB)

Supplementary file9 (TIF 2530 KB)

## Data Availability

All data related to this work is presented in the paper and its supplements. Raw data is available at https://doi.org/10.5061/dryad.vx0k6djpp. The materials used in this study are available to any qualified researcher upon reasonable request addressed to D.M.H.

## Code availability

The code for network activity simulations is provided as a supplement to this paper. This code can be freely modified or re-used for non-commercial purposes if appropriately cited. The Synapse counter plugin is available at https://github.com/SynPuCo/SynapseCounter. All minor codes for image analysis are available upon reasonable request addressed to E.D.
