# Supplementary figures and images for: Inhibitory control in neuronal networks relies on the extracellular matrix integrity
Source: Cell Mol Life Sci. 2021 Jun 15;78(14):5647–63. doi: 10.1007/s00018-021-03861-3 (PMC8257544; doi:10.1007/s00018-021-03861-3)

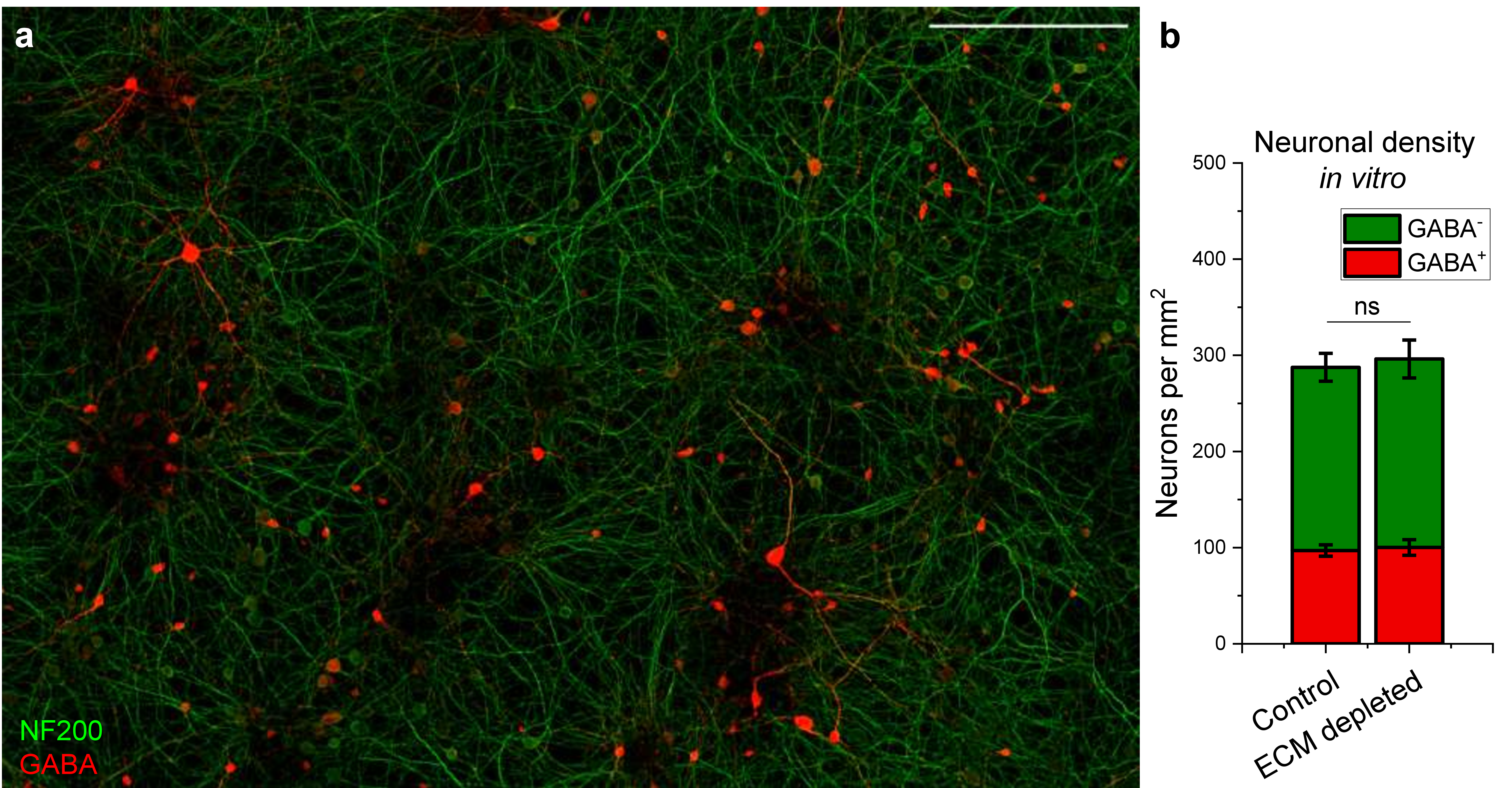

Supplement: Supplementary file 2 — Supplementary file2 (TIF 59900 KB) [file 18_2021_3861_MOESM2_ESM.tif]

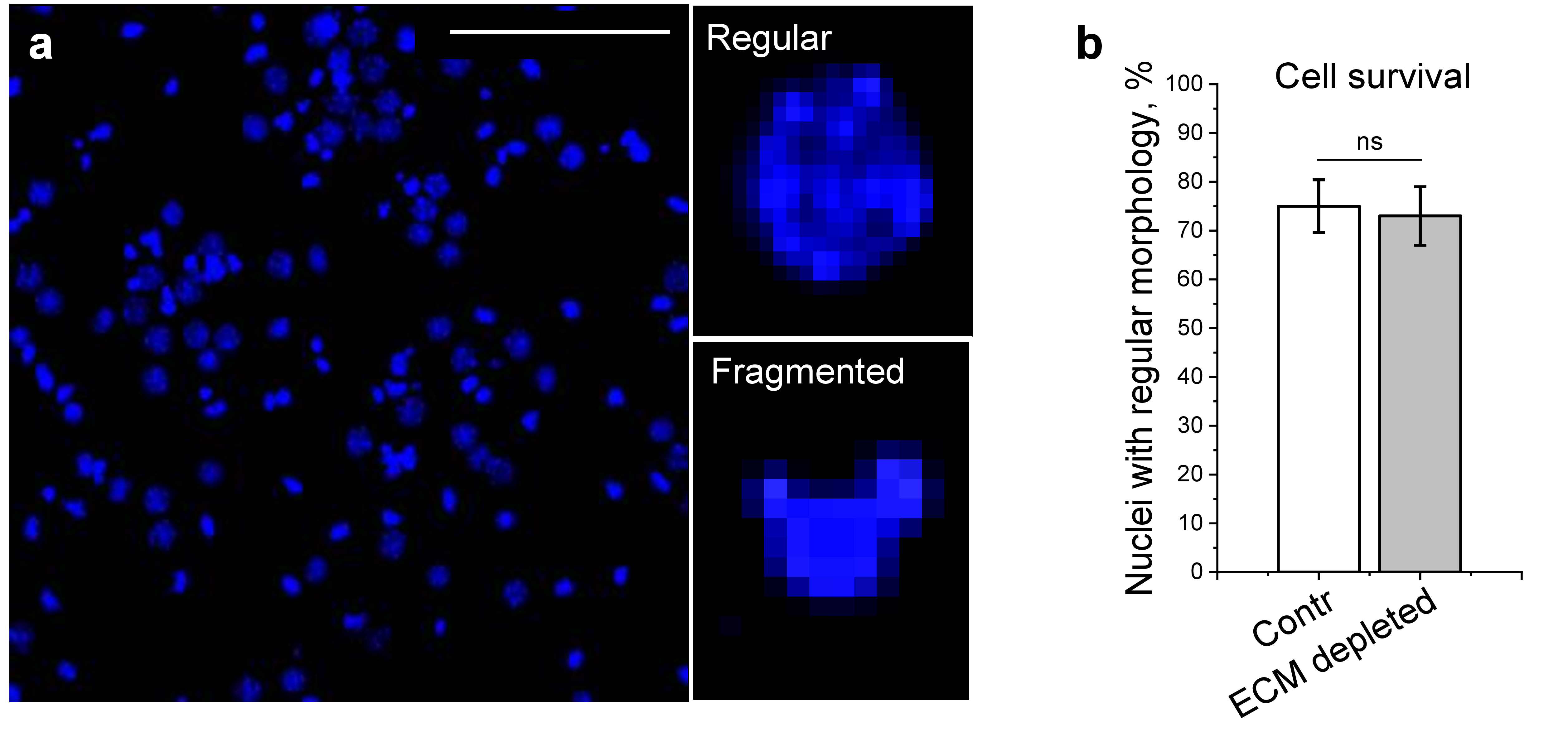

Supplement: Supplementary file 5 — Supplementary file5 (TIF 34823 KB) [file 18_2021_3861_MOESM5_ESM.tif]

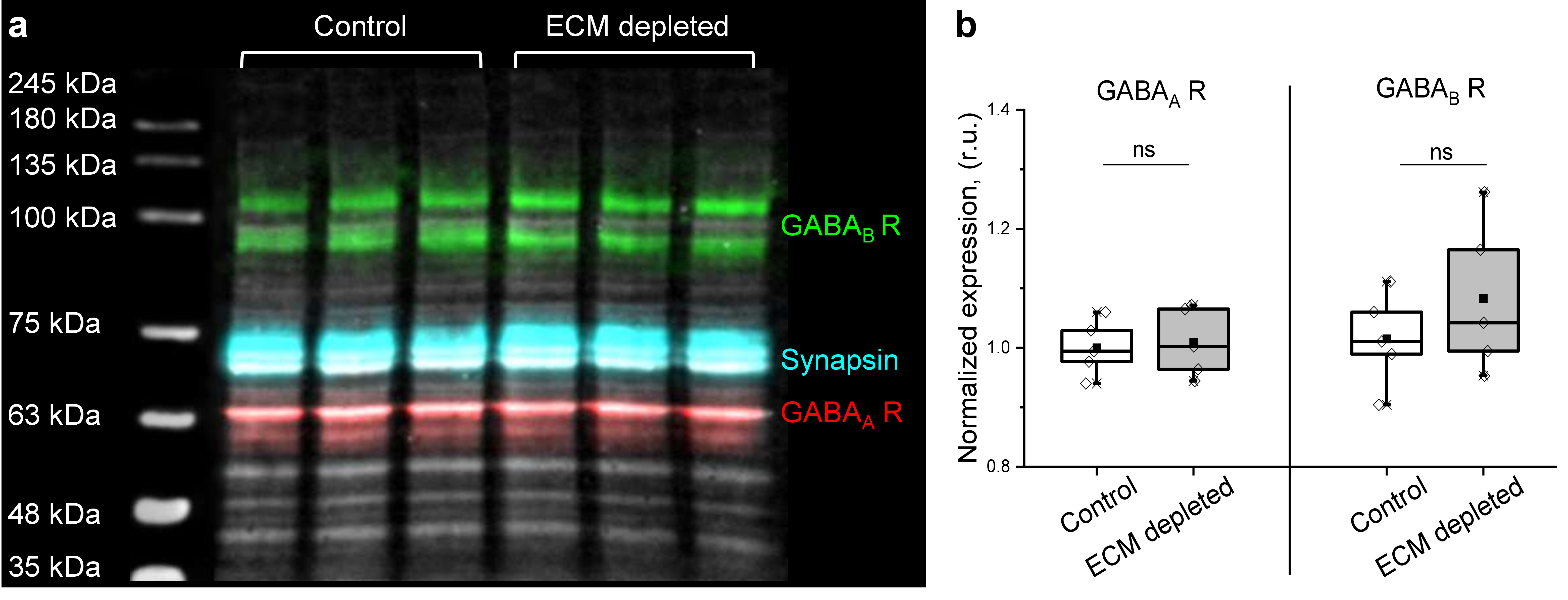

Supplement: Supplementary file 6 — Supplementary file6 (TIF 43656 KB) [file 18_2021_3861_MOESM6_ESM.tif]

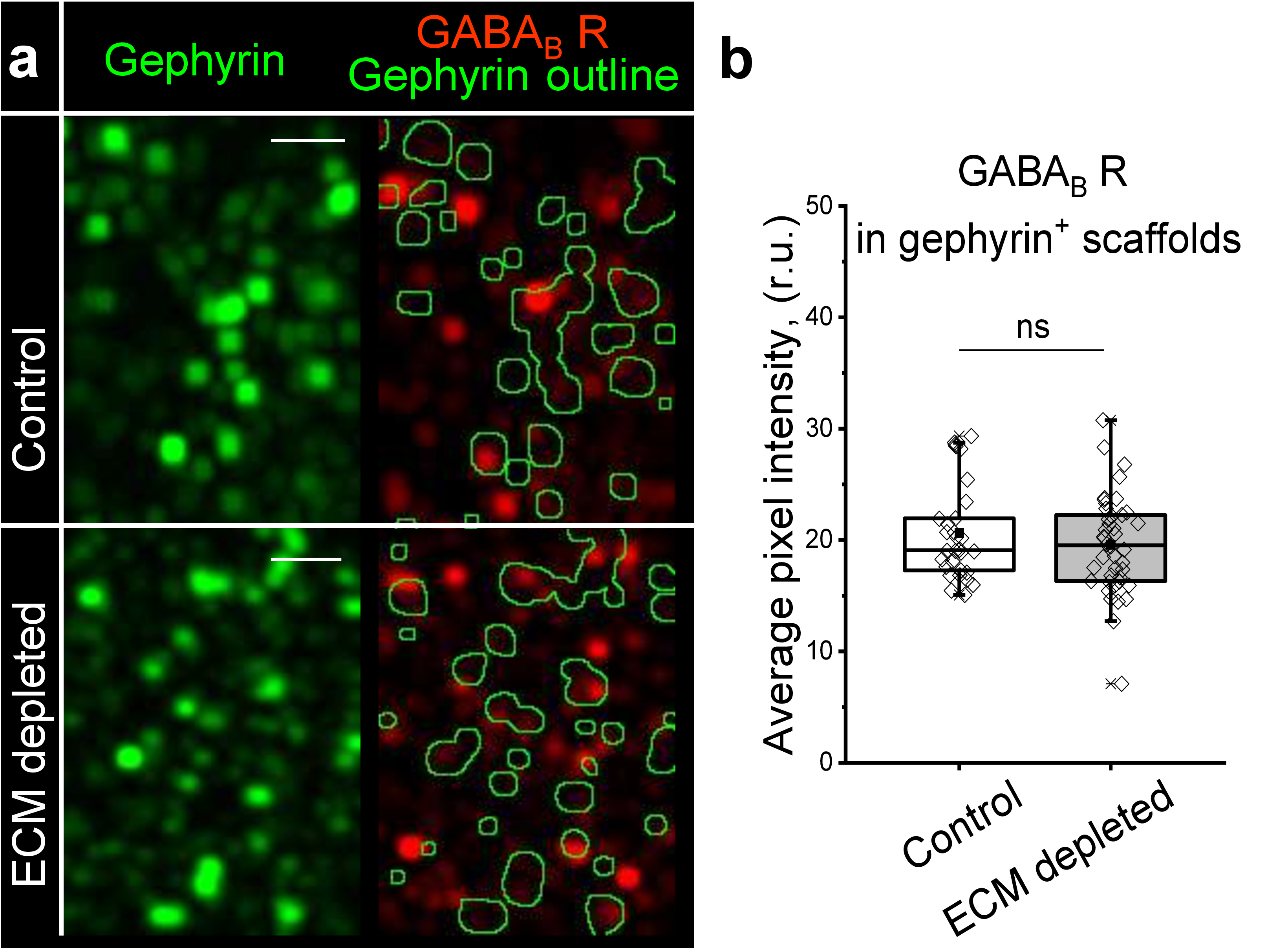

Supplement: Supplementary file 7 — Supplementary file7 (TIF 23094 KB) [file 18_2021_3861_MOESM7_ESM.tif]

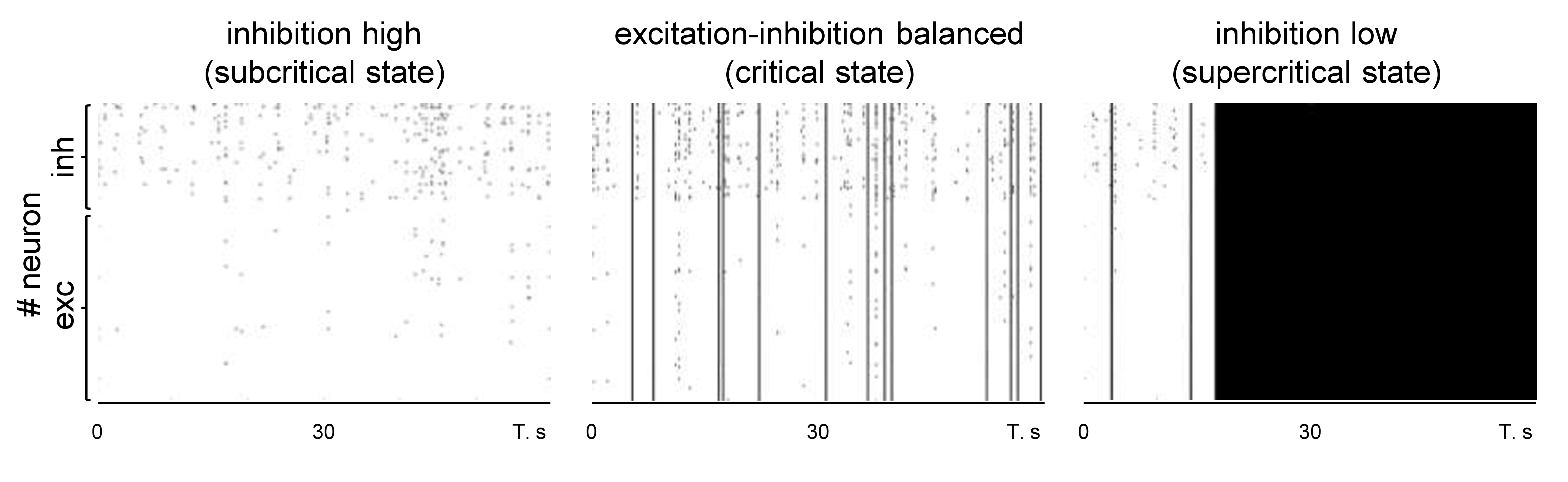

Supplement: Supplementary file 8 — Supplementary file8 (TIF 2355 KB) [file 18_2021_3861_MOESM8_ESM.tif]

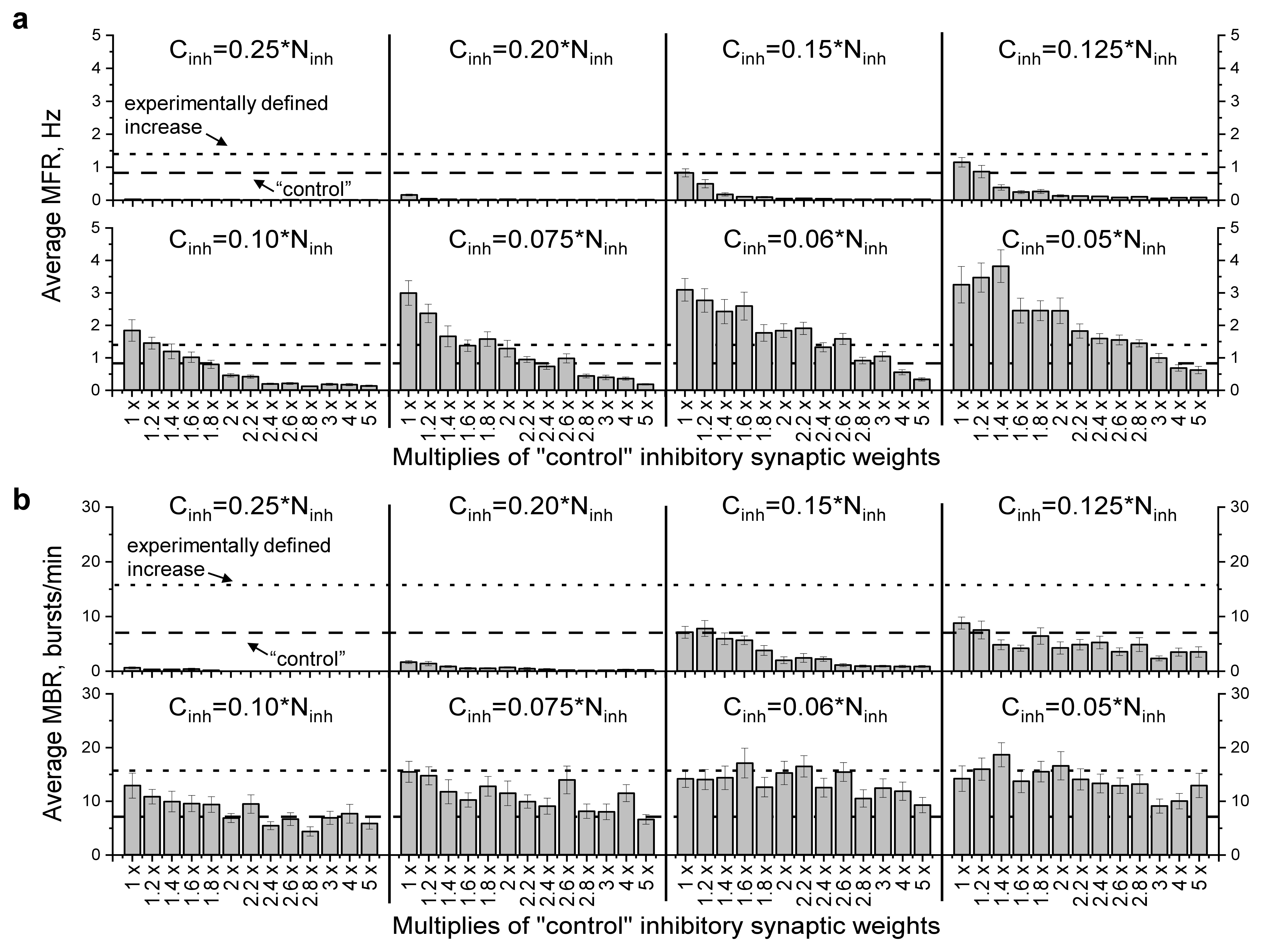

Supplement: Supplementary file 9 — Supplementary file9 (TIF 2530 KB) [file 18_2021_3861_MOESM9_ESM.tif]
